# Parallel processing of T cells and natural killer cells to enhance *in vivo* CAR T cell activity against blood and solid cancers

**DOI:** 10.1002/cti2.70120

**Published:** 2026-09-01

**Authors:** Lachlan J Dobson, Patrick W Marron, Ariana HT Drabble, Thiloma D Liyanage, Kristine C Pe, Clare Y Slaney, Barry D Hock, Alexander D McLellan

**Affiliations:** ^1^ Department of Microbiology and Immunology The University of Otago Dunedin New Zealand; ^2^ Charles Perkins Centre, Faculty of Medicine and Health, School of Medical Sciences University of Sydney Camperdown NSW Australia; ^3^ Cancer Immunology Program Peter MacCallum Cancer Centre Melbourne VIC Australia; ^4^ Sir Peter MacCallum Department of Oncology University of Melbourne Parkville VIC Australia; ^5^ Haematology Research Group, Department of Pathology and Biomedical Science University of Otago Christchurch New Zealand; ^6^ Haematology Department Christchurch Hospital Christchurch New Zealand; ^7^ Department of Histology, Faculty of Medicine University of Indonesia Jakarta Indonesia

**Keywords:** cell therapy, chimeric antigen receptor, manufacturing, natural killer, T cell

## Abstract

**Objectives:**

Despite their anti‐cancer properties, natural killer (NK) cells are discarded during CAR T cell manufacturing. NK cells display promising responses as a cell therapy, exhibiting remarkable safety at high doses. Moreover, NK cells have complementary cytokine profiles and distinct antigen‐recognition pathways to supplement CAR T cell therapies. We demonstrate parallel expansion of NK cells from the normally discarded PBMC of CAR T cell production for downstream combination therapies against solid and blood‐borne malignancies.

**Methods:**

CAR T cells were manufactured from buffy coats with initial isolation of T cells from PBMC. NK cells were rapidly expanded in parallel by stimulating the remaining PBMC with membrane‐bound (mb)IL‐21 + mbIL‐15 feeder cells. Expanded NK cells were assessed through spectral flow cytometry and applied as a pretreatment prior to CAR Tcell infusion against xenograft tumor models in NOD‐*scid* IL2Rgamma^null^ (NSG) mice.

**Results:**

Applying feeder cells to residual PBMC stimulated 5000‐fold expansions of pure NK cells. Expanded NK cells expressed various activation receptors and displayed strong *in vitro* cytotoxicity, which was further improved in CAR T‐conditioned medium. A pretreatment of NK cells enhanced existing CAR T cell therapies, improving *in vivo* tumor clearance and survival against breast cancer, acute lymphoblastic leukaemia and non‐Hodgkin's lymphoma. While NK cells eliminated CD19^−/−^ variants *in vitro*, they were unable to suppress antigen‐escape relapse *in vivo*.

**Conclusion:**

This study encourages the optimal use of leukapheresis product. By recycling discarded PBMC into NK cells, CAR T cell therapies can be effectively enhanced, improving tumor clearance and survival.

## Introduction

Equipping T cells with chimeric antigen receptors (CARs) has revolutionised the treatment of blood‐borne malignancies. With the addition of Aucatzyl for ALL in 2024, there are now seven FDA‐approved CAR T cell products for the treatment of either multiple myeloma or B cell lymphoma/leukaemia.^1^, ^2^ CAR T cells are now an effective treatment option for patients with relapsed/refractory disease, though they only achieve complete remission in a subset of patients. Indeed, CAR T cells are limited by suppressive tumor microenvironments and become redundant when malignant cells down‐regulate the target antigen.^3^, ^4^, ^5^

Natural killer (NK) and CAR NK cell therapy is an emerging cellular therapy with promise to expand the range of antigens targeted.^6^, ^7^, ^8^ NK cells can exert immediate cytotoxicity against malignancies via target recognition of a range of germ‐line‐encoded receptors binding stress/activatory ligands, as well as recognising the absence of MHC class I.^8^ Due to the lack of diverse T cell receptors, NK cells display a more limited reaction to mismatched HLA alleles and can be administered in an off‐the‐shelf, allogeneic setting,^6^, ^7^, ^8^ particularly following induction patient conditioning. A toxic dose of CAR NK cells has not been noted, likely due to a distinct cytokine profile that deviates from classic CRS.^9^ However, while allogeneic CAR NK cells display remarkable safety, their translation in clinical trials is hindered by host rejection^10^, ^11^ in addition to low persistence and dysfunction impacted by limiting levels of gamma‐chain cytokines, including IL‐2 or IL‐15.^12^, ^13^

While CAR T and NK cells show efficacy in their respective monotherapies, it is likely that autologous NK and CAR T cells would synergise for an enhanced combination therapy. NK cells can recognise a range of tumor‐associated factors to supplement the mono‐antigen outlook of a CAR T cell, while CAR T cells naturally secrete IL‐2 upon activation to theoretically sustain an NK cell response. In addition, CAR NK cells destroy myeloid‐derived suppressor cells to facilitate subsequent invasion of CAR T cells in solid tumors,^14^ while improving CAR T cell fitness in non‐Hodgkin's lymphoma.^15^ We and others have noted that NK cells can act as helper cells for pMHC‐specific cytotoxic T cells, enhancing therapy in a mouse model of melanoma.^16^, ^17^

During CAR T cell manufacturing, T cells are isolated from the leukapheresis product while the remaining PBMC are discarded. Due to the proven efficacy of NK cell treatments and the complementary nature of NK and CAR T cells, we developed a protocol for parallel expansion of NK and CAR T cells from the same PBMC product. In‐house prepared feeder cells were used to derive activated NK cells from residual PBMC to enable combination therapy to begin at least 2 days prior to administration of CAR T cells. NK cell pretreatment significantly enhanced existing CAR T cell therapies against both solid and blood cancers, justifying exploration of future combination cell therapies.

## Results

### Neglected PBMC of CAR T cell manufacturing can be repurposed for expanded NK cell product

**Figure 1:**
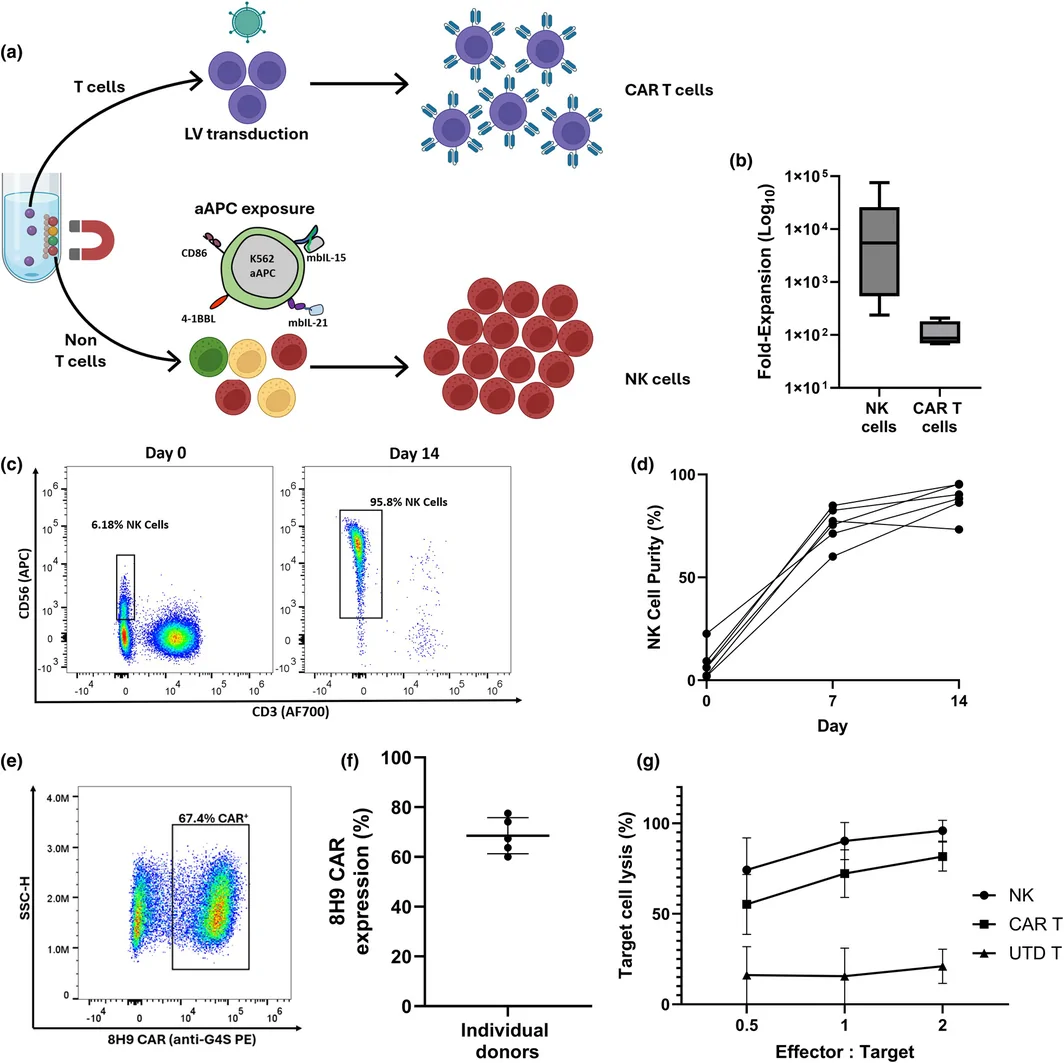
Natural killer (NK) cells can be readily derived from residual PBMC in CAR Tcell manufacturing. T cells were isolated from PBMC with a negative‐selection kit and transduced, while the bead‐bound non‐T cells were recycled and stimulated with artificial antigen presenting cells (aAPC) for NK cell expansion **(a)**. PBMC receiving a 1:1 single dose of feeder cells exhibited 5000‐fold expansions, while CD3/28‐activated CAR T cells expanded by 100‐fold in 14 days **(b)**. aAPC‐expanded products displayed highly pure populations of NK cells **(c, d)**. T cells were readily transduced, attaining high expressions of 8H9 CAR **(e, f)**. Both NK cells and CAR T cells displayed high cytotoxicity against MCF‐7 cells when applied at 0.5:1, 1:1 and 2:1 effector‐to‐target ratios **(g)**. Fold expansions displayed as mean (with interquartile range) with whiskers ranging from minimum to maximum, (*n* = 4–6). Cytotoxicity displayed as mean ± SD, (*n* = 3). Cytotoxicity assays carried out in R10 supplemented with 50 U/mL IL‐2.

For manufacturing CAR T cells, T cells were isolated from PBMC with a negative‐selection protocol.^18^ While non‐T PBMC are generally discarded, we collected these cells and initiated NK cell expansion using our feeder cell system (Figure 1a). Non‐T PBMC were stimulated at a 1:1 ratio with irradiated aAPC expressing mbIL‐21, mbIL‐15, 4‐1BBL and CD86. Upon aAPC exposure, NK cells were selectively expanded, with 5000‐fold expansion apparent at 14 days (Figure 1b). The expanded cells were enriched for NK cells, with the median population containing 89.3% NK cells (*n* = 6, range 73.3–95.4%) (Figure 1c and d). Prior to expansion, NK cells were largely of the CD56^dim^, CD16^+^ subtype; however, by Day 7, the NK cells had differentiated into a dual‐positive, CD56^bright^, CD16^+^ phenotype (Supplementary figure 1).

During NK cell expansion, the isolated T cells were expanded and transduced with 8H9 CAR, targeting B7‐H3^19^ (Figure 1b, e and f). Individually, the NK cells and CAR T cells exhibited potent cytotoxicity towards MCF‐7 breast cancer cells at 0.5:1, 1:1 and 2:1 effector‐to‐target ratios (Figure 1g).

### Recycled NK cells express an array of activation receptors

**Figure 2:**
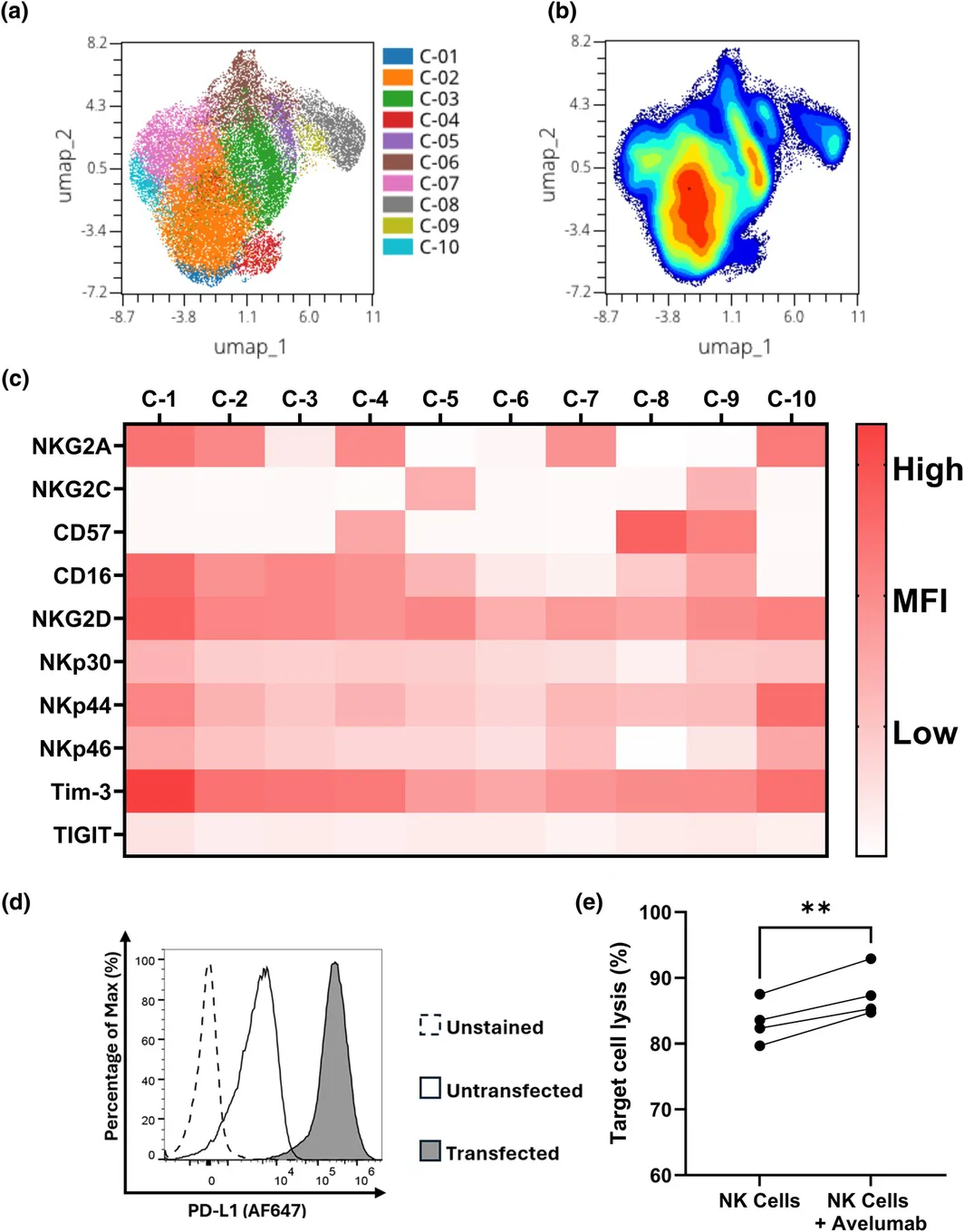
Phenotypic characterisation of expanded natural killer (NK) cells. NK cells were characterised on Day 14 of expansion. Uniform Manifold Approximation and Projection (UMAP) plots show the 10 distinct meta clusters of NK cells generated via FLOWSOM **(a)**, with a contour UMAP indicating proportions of NK cells belonging to each cluster **(b)**. A clustered heatmap visualisation displays the MFI of each indicated marker belonging to NK cells within the corresponding clusters **(c)**. To model ADCC, PD‐L1 was over‐expressed on MCF‐7 cells **(d)** prior to cytotoxicity assay in the presence/absence of 10 μg/mL avelumab **(e)**. NK Cell phenotyping, *n* = 3. Cytotoxicity displayed as individual donors (*n* = 4). **P ≤ 0.005, by multiple paired *t*‐tests.

We assessed the phenotypes of expanded NK cells with high‐dimensional analysis, paying particular attention to receptors for tumor recognition. NK cells (CD3^−^, CD56^+^) were subsampled from the expanded product and subjected to Flow SOM and consensus meta clustering using the markers in Figure 2c. Ten cell clusters were generated based on variable expressions of markers and visualised using UMAP (Figure 2a). An accompanying contour plot (Figure 2b) shows that most NK cells correspond to Clusters 2 and 3. These NK cells are primed for cancer recognition, with high expression of CD16, NKG2D, NKp30, NKp44 and NKp46^20^ (Figure 2c). Other, less frequent populations include Clusters 5 and 9, corresponding to adaptive ‘memory‐like’ NK phenotypes through expression of NKG2C.^20^ Throughout the 14‐day expansions, the frequencies of NKG2A^+^ and NKG2C^+^ NK cells remained consistent with neither subset being preferentially expanded or displaying significant changes in frequency following expansion (Supplementary figure 2).

To devise a model of ADCC, we over‐expressed the immune checkpoint inhibitor, PD‐L1, on MCF7 cells (Figure 2d). Tumor cells were coated with the anti‐PD‐L1 IgG_1_, avelumab, prior to incubation with NK cells at a 1:1 effector‐to‐target ratio.

While NK cells displayed potent cytotoxicity, the additional pretreatment with avelumab improved cytotoxicity, reflecting the CD16 expression of expanded NK cells (Figure 2e).

### CAR T cell activity supports NK cell responses

Despite extensive innate sensing of tumor cells, adoptively transferred NK cells are strongly reliant on gamma‐chain cytokines for sustained activity. Indeed, *in vivo* models often rely on ectopic NK cell cytokine production or knocking‐in supporting cytokines into the mice.^21^, ^22^, ^23^ It is well known that CAR T cells release IL‐2, IFN‐γ and TNF‐α upon antigen recognition.^15^ We hypothesised that activated CAR T cells would secrete soluble factors to support NK cell responses, while unsupported NK cells would display limited activity.

Testing this hypothesis, we cultured NK cells in media without cytokine support to approximate *in vivo* NSG conditions during NK cell monotherapy. To replicate the conditions of combination therapy, NK cells were cultured in autologous, tumor‐activated CAR T cell supernatant (CSN). As a control, NK cells were also cultured in their conventional IL‐2‐supplemented media (50 U/mL).

The CAR T cell supernatant was analysed through mass spectrometry and contained an extensive array of soluble factors, including IL‐2, TNF, IFN‐γ, CXCL9, CXCL10, CCL1 and CCL2 (Supplementary figure 4a–c). Activated NK cell supernatant was similarly analysed as a comparison, containing similar proteins, though at a lower measured abundance. By ELISA analysis, the IL‐2 in the CAR T CSN was quantified, with a mean readout of 25 ng/mL (range: 17.7–40.0)(Supplementary figure 4b).

**Figure 3:**
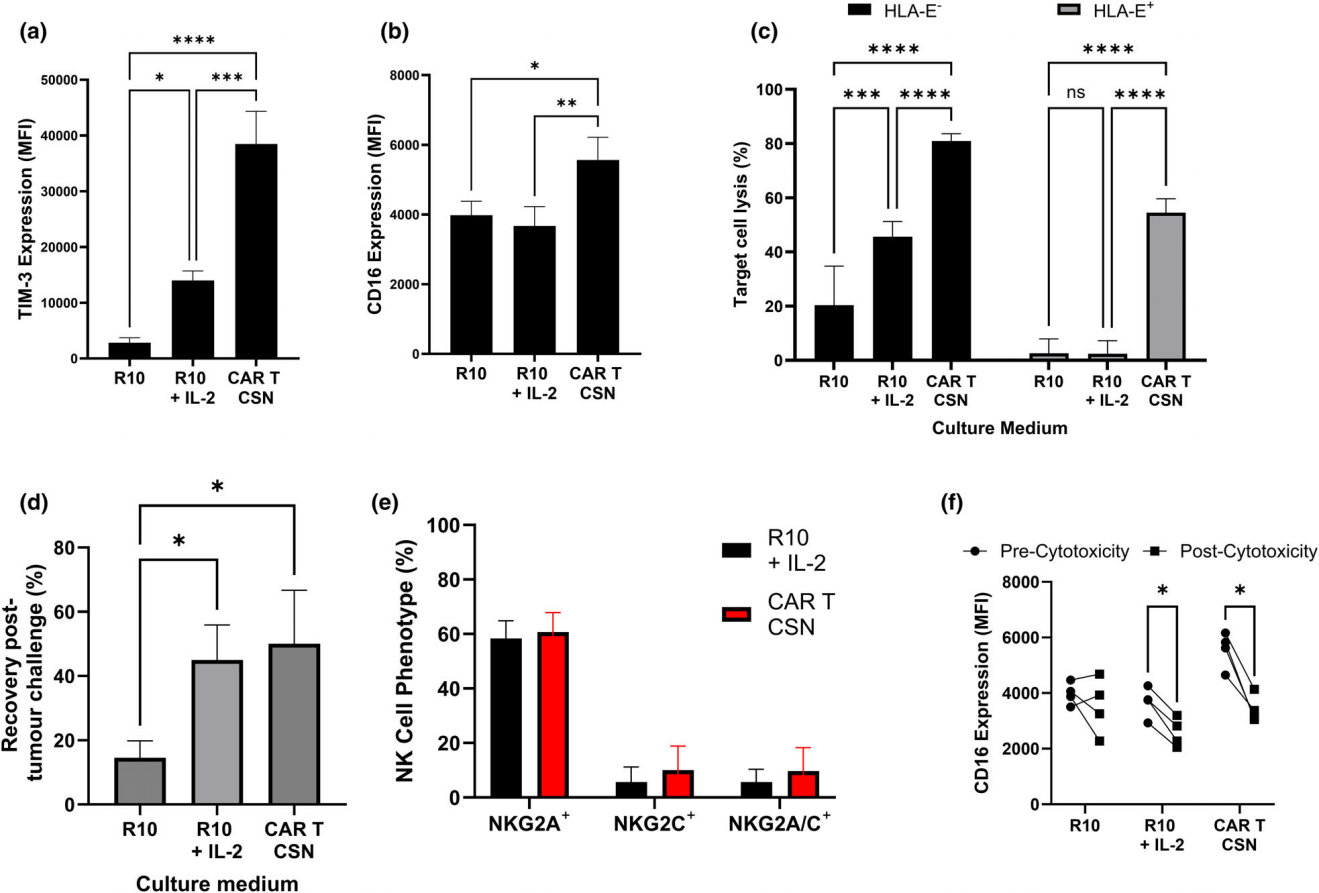
Mutual benefits of natural killer (NK) and CAR T cells. NK cells were cultured for 48 h in either R10, R10 + 50 U/mL IL‐2 or in antigen‐activated CAR T cell supernatant (CSN). Culturing NK cells in activated CAR T cell supernatant heightened expression of TIM‐3 and CD16 **(a, b)**. CAR T CSN‐stimulated NK cells displayed improved lysis of both HLA‐E^−^ and HLA‐E^+^ tumor cells at a 0.5:1 effector‐to‐target ratio **(c)** and improved recovery following cytotoxicity **(d)**. The frequency of NKG2A and NKG2C NK cells was consistent across the different culture media **(e)**. Culture within CAR T CSN was unable to rescue NK cells from CD16‐cleavage following cytotoxicity **(f)**. (*n* = 4 individual donors. Data displayed as mean + SD. *P ≤ 0.05, **P ≤ 0.005, ***P ≤ 0.0005, ****P < 0.0001 by two‐way ANOVA) with Bonferroni correction. CD16 cleavage displayed for individual donors, *P ≤ 0.05 by multiple paired *t*‐tests.

After 2 days of culture, we observed increased expression of CD16 and TIM‐3, alongside potent cytotoxicity from CAR T CSN‐stimulated NK cells against MCF‐7 targets at a 0.5:1 effector‐to‐target ratio (Figure 3a–c). Comparatively, cytokine‐starved NK cells were unable to effectively lyse targets. In addition to rejuvenated cytotoxicity, CAR T CSN‐stimulated NK cells were able to maintain higher populations following cytotoxicity than cytokine‐starved NK cells (Figure 3d), supporting the hypothesis that CAR T cell activation is vital to NK cell function in combination therapies.

In addition to strong lysis of conventional, HLA‐E^−^ MCF‐7 cells, CAR T CSN‐activated NK cells were able to overcome HLA‐E‐overexpressing MCF‐7 cells, whereas cytokine‐starved, or IL‐2 stimulated NK cells elicited no response (Figure 3c). No changes in NKG2A or NKG2C populations were observed upon addition of CAR T CSN (Figure 3e). While CAR T cells provided conditions for heightened NK cell activity, NK cells were not rescued from cleaving CD16 post‐cytotoxicity (Figure 3f).

### A pretreatment of recycled NK cells enhances CAR T cell therapy for immunosuppressive solid tumors and acute lymphoblastic leukaemia

**Figure 4:**
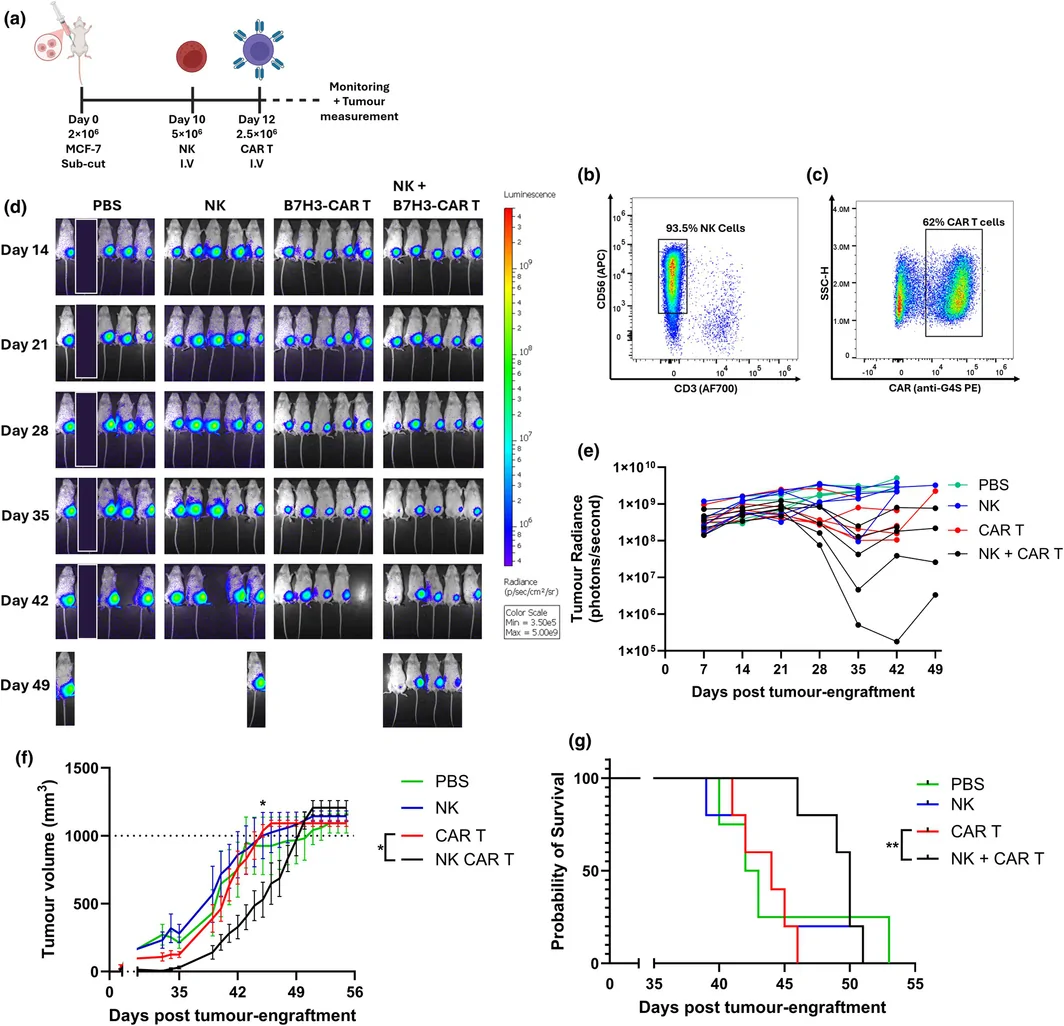
Recycled natural killer (NK) cells enhance CAR T cell therapy of MCF‐7. NSG mice were engrafted with Her2^h^ ^i^ PDL1^hi^ MCF‐7 cells on Day 0 prior to sequential administration of NK cells (d10) and CAR T cells (d12) **(a)**. Quality control of both cell types ensured high NK cell purity and transduction efficiency of T cells prior to administration **(b, c)**. Mice were imaged weekly **(d, e)**, and callipers were used every other day to monitor tumor burden **(f)**. Mice were euthanised when radiance and/or tumor volume reached endpoint thresholds **(g)**. Data from one mouse experiment. *n* = 4–5 mice per group. Tumor burdens displayed as mean ± SEM. *P < 0.05 by two‐way ANOVA with Bonferroni correction. Survival analysed with Kaplan–Meier, log‐rank mantel‐cox test. **P ≤ 0.005. White out‐lined box conceals mouse removed from study because of failed tumor engraftment.

For solid‐tumor generation, NOD‐*scid* IL2Rgamma^null^ (NSG) mice were subcutaneously injected in their left abdominal mammary fat pads with 2 × 10^6^ MCF‐7 cells overexpressing PD‐L1, HER‐2 and luciferase. By employing our parallel‐expansion technique, separate NK cell and CAR T cell products were generated from a single PBMC preparation and engrafted into mice without prior cryopreservation. Previous studies have identified that NK cell pretreatments can improve CAR T cell infiltration into solid tumors through the secretion of T cell‐recruiting chemokines.^14^, ^24^, ^25^ Therefore, mice received intravenous injections of 5 × 10^6^ NK cells on Day 10 post tumor‐engraftment and 2.5 × 10^6^ CAR T cells on Day 12 (Figure 4a). The CAR T cell dose was identified as non‐curative in a separate pilot study (Supplementary figure 3). Mice were treated with either the full treatment regime or separate monotherapies of either NK cells or CAR T cells. Mice in control groups received PBS in place of missing treatments. Day 10 NK cell purity was 91.2% while CAR T cells displayed 62% CAR expression on Day 12 (Figure 4b and c).

The monotherapy with expanded NK cells resulted in no tumor control, while a monotherapy of CAR T cells plateaued tumor growth, with only slight reductions in tumor radiance (Figure 4d and e), and no observed suppression of tumor outgrowth (Figure 4f). While tumors overcame the singular therapies, CAR T cells were enhanced with a pretreatment of NK cells, improving overall tumor control (Figure 4d–f). As a result, median survival increased from 44 days in CAR T cell‐treated mice to 50 days upon the addition of NK cells (*P* = 0.0044) (Figure 4g). Mice treated with CAR T alone succumbed to tumor burden at over 14‐fold (hazard ratio) the rate of those treated with combination of NK cells and CAR T cells. In clinical translation, a single, combined dose of NK and CAR T cells would improve logistics of the therapy by allowing for a single round of batch‐release testing and a single infusion. However, retrospective testing identified that co‐administrating NK and CAR T cells ablated the benefits of the combination therapy, leading to significantly decreased tumor clearance (Supplementary figure 5).

**Figure 5:**
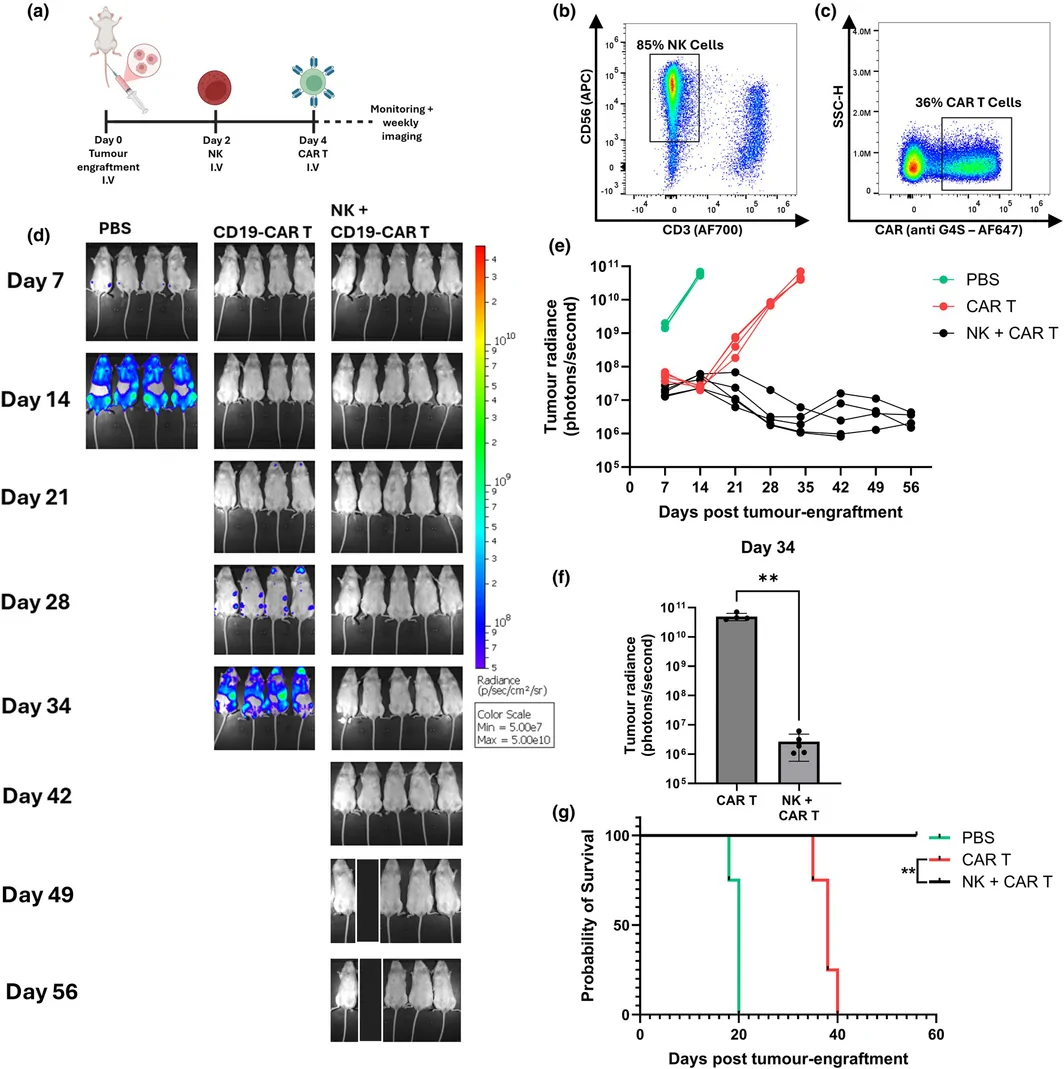
Natural killer (NK) cell pretreatment prior to CAR T cells enables for clearance of acute lymphoblastic leukaemia. NSG mice were engrafted with NALM6 cells on Day 0 prior to sequential administration of NK cells (d2) and CAR T cells (d4) **(a)**. Quality control of both cell types ensured high NK cell purity and transduction efficiency of T cells prior to administration **(b, c)**. Mice were imaged weekly **(d–f)** and monitored daily for behavioural changes. Mice were euthanised when radiance and/or behaviours reached endpoint thresholds **(g)**. Data from one mouse experiment. *n* = 4–5 mice per group. Tumor burdens displayed as mean ± SD. **P < 0.005 by paired *t*‐test. Survival analysed with Kaplan–Meier, log‐rank mantel‐cox test. **P ≤ 0.005. White out‐lined box conceals mouse removed from study because of a non‐tumor‐related event.

The NK cell recycling and pretreatment regime was adapted for an acute lymphoblastic leukaemia model. NSG mice were intravenously engrafted with 1 × 10^6^ NALM6‐luciferase cells on Day 0 prior to 5 × 10^6^ NK cells on Day 2 and 2.5 × 10^6^ CD19‐CAR T cells on Day 4 (Figure 5a). NK and CAR T cell purities were confirmed with flow cytometry (Figure 5b and c) prior to infusion. While the CD19 CAR T cells displayed initial clearance of Nalm‐6, tumor burden increased post‐Day 14 (Figure 5d and e), leading all mice to endpoint by Day 40 (Figure 5g). In contrast, the addition of NK cells maintained significantly lower levels of tumor cells, displaying an 18 000‐fold reduction in tumor radiance compared to CAR T only on Day 34 (Figure 5d–f) and prolonging survival until the cessation of the experiment (Figure 5g).

### NK cells persist after tumor clearance and promote a distinct T cell phenotype

The NK and CAR T cell combination therapy was further tested against MCF‐7 with parental expression of PD‐L1 as opposed to the over‐expressed line utilised in Figure 4. Without the overexpression of PD‐L1, the MCF‐7 cells were fully cleared by the combination therapy by Day 20 post‐CAR T cell infusion (Supplementary figure 6). The monotherapy of CAR T cells similarly cleared the tumors, however, at a delayed rate.

**Figure 6:**
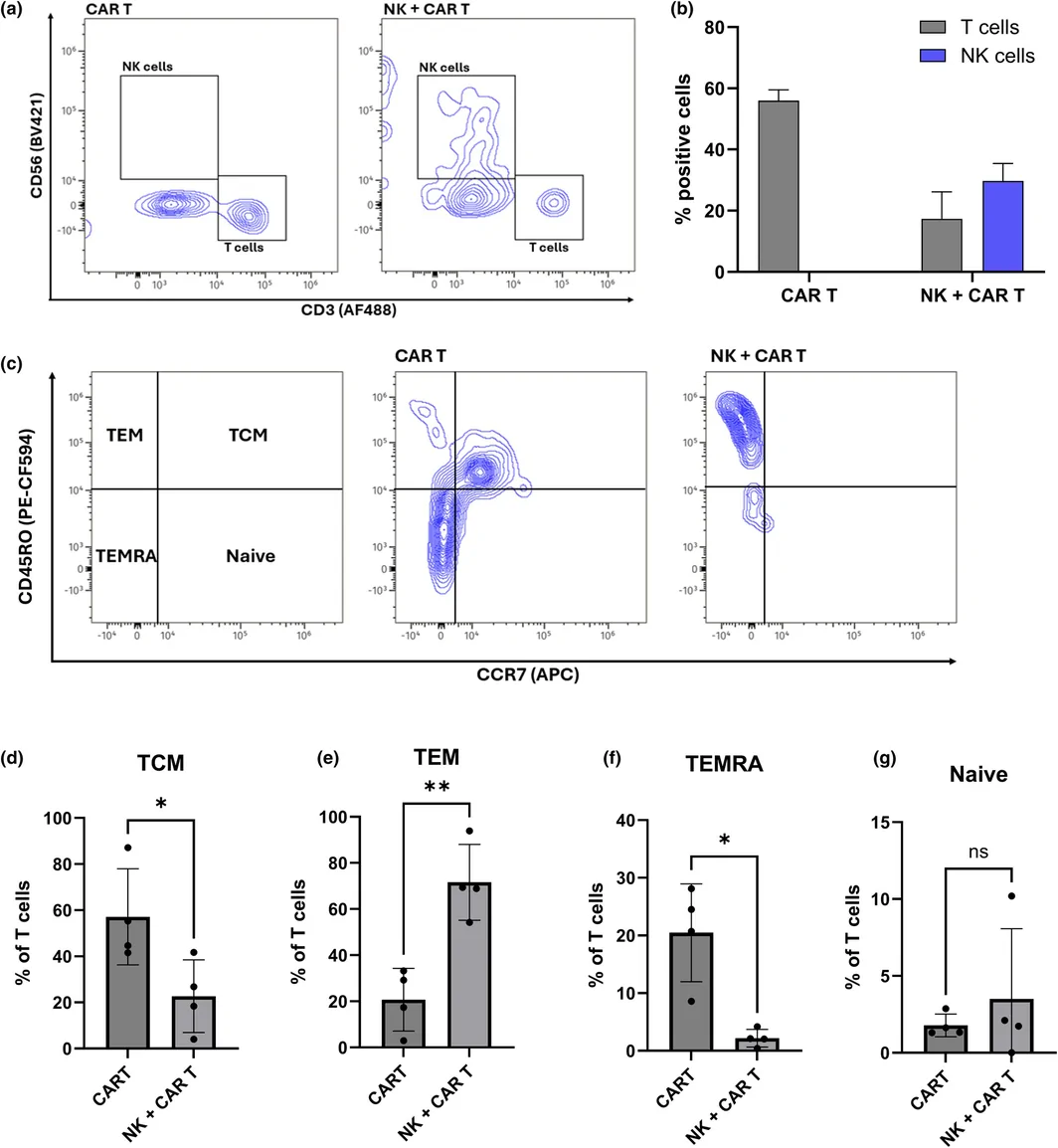
Analysis of splenic natural killer (NK) and CAR T cells following tumor clearance. After clearing an MCF‐7 solid tumor, spleens were harvested from NSG mice and analysed for cell types and T cell phenotype. By staining for CD3 and CD56, distinct populations of T and NK cells were identified **(a, b)**. The T cells were further stained for CCR7 and CD45Ro and classified into phenotypes **(c)** to deduce frequencies of central memory (TCM), effector memory (TEM), terminally differentiated effector memory (TEMRA) and naïve T cells **(d–g)**. Data from one mouse experiment. *n* = 4 mice per group. Flow plots display one representative donor. NK and T cell frequencies displayed as mean ± SEM. T cell phenotypes displayed as mean ± SD and analysed by unpaired *t*‐test in which *P < 0.05, ** *P* < 0.005.

Mice were euthanised on Day 70 and spleens were harvested and processed for flow cytometric analysis. Staining for CD3 and CD56 revealed that both NK and T cells persisted following the tumor regression (Figure 6a and b). T cells were subsequently analysed for the expression of CD45RO and CCR7 and assigned respective maturation phenotypes (Figure 6c). Compared to administering CAR T cells alone, the combined therapy decreased the percentage of central memory T cells (TCM), while driving a higher differentiation into effector memory T cells (TEM) (Figure 6d and e). In addition, the presence of NK cells decreased the terminal differentiation of T cells (TEMRA), maintaining T cells with effector memory phenotypes (Figure 6f). No impact on naïve T cell frequencies was observed (Figure 6g).

### Equipping recycled NK cells with CAR further improves therapy against non‐Hodgkin's lymphoma

In parallel with the ALL study, we sought to translate the combination therapy to a model of non‐Hodgkin lymphoma (NHL). Additionally, we aimed to improve NK cell activity by expressing a CD20 CAR to supplement the CD19 CAR T cells. During NK cell transductions, use of an EF1a promoter resulted in minimal CAR expression. This was improved by incorporating an MSCV promoter (Supplementary figure 7).

**Figure 7:**
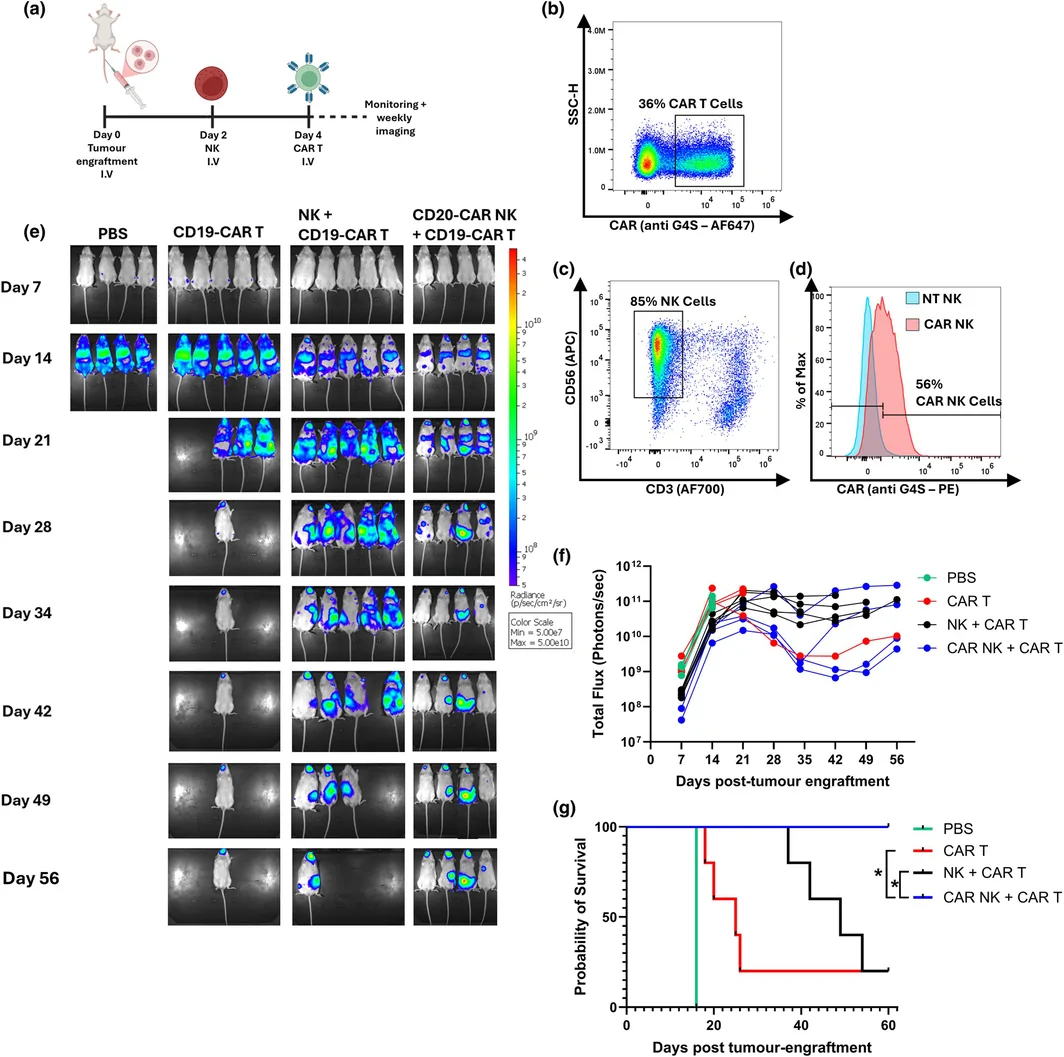
Natural killer (NK) cell pretreatments can be further improved with CAR expression prior to dual‐antigen targeting with CAR T cells. NSG mice were engrafted with Raji cells on Day 0 prior to sequential administration of NK/CAR NK cells (d2) and CAR T cells (d4) **(a)**. Quality control of both cell types ensured high transduction efficiency of NK and T cells prior to administration **(b–d)**. Mice were imaged weekly **(e, f)** and monitored daily for behavioural changes. Mice were euthanised when radiance and/or behaviours reached endpoint thresholds **(g)**. Data from one mouse experiment. *n* = 4–5 mice per group. Survival analysed with Kaplan–Meier, log‐rank mantel‐cox test. *P ≤ 0.05.

To model NHL, mice received 1 × 10^6^ Raji‐luciferase cells (IV) on Day 0, prior to 4 × 10^6^ NK cells on Day 2 and 2 × 10^6^ CAR T cells on Day 4 (Figure 7a). Cell purities and CAR expression were measured prior to infusion, with approximately 56% of transduced NK cells expressing CD20 CAR (Figure 7b–d). Mice receiving CAR T cells alone had rapid tumor progression, succumbing in under 30 days (Figure 7e–g). Addition of NK cells improved tumor control and prolonged median lifespan from 26 to 49 days. Equipping NK cells with CD20 CAR further enhanced tumor clearance and all mice survived until experiment cessation (Figure 7e–g).

### Co‐administered NK cells cannot prevent antigen‐escape relapse during CAR T cell treatments

**Figure 8:**
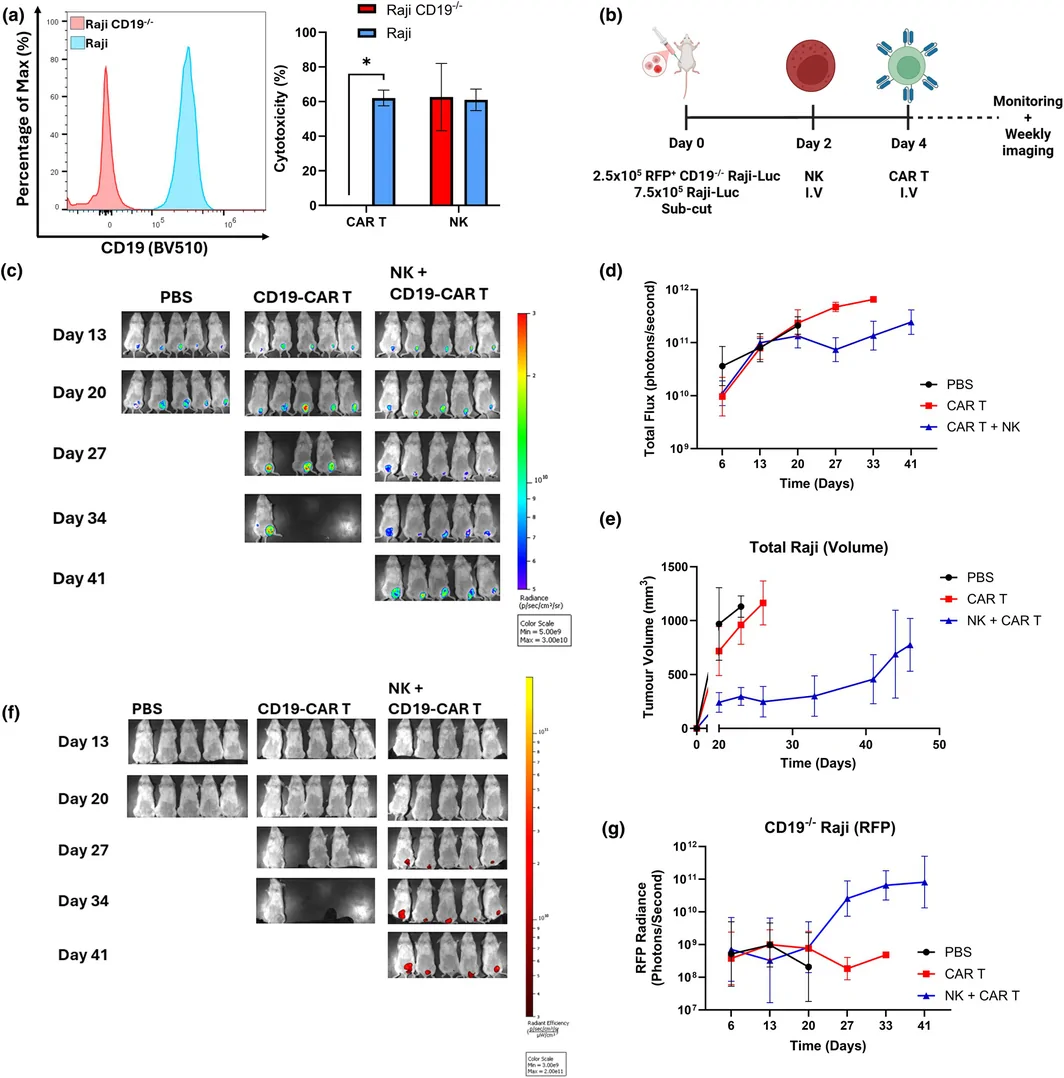
Natural killer (NK) cells can lyse antigen‐negative cells *in vitro*, but do not prevent relapses of antigen‐negative tumors in xenografts. **(a, b)** NK cells and CAR T cells were applied to Raji cells with or without expression of CD19 **(a)** at a 1:1 effector‐to‐target ratio. While CAR T cells relied on CD19 expression for activity, NK cells lysed Raji in the presence and absence of CD19. For the xenograft experiment, NSG mice were subcutaneously engrafted with a mixture of 7.5 × 10^5^ CD19^+^ and 2.5 × 10^5^ CD19^−^ Raji‐Luc cells on Day 0. CD19^−^ Raji cells simultaneously expressed RFP. Mice were subsequently administered with 2 × 10^6^ NK cells on Day 2 and 1 × 10^6^ CAR T cells on Day 4 **(b)**. Mice were imaged weekly and assessed for luciferase activity **(c, d)** and for RFP fluorescence **(f, g)**. Mice were also assessed for physical tumor volume through calliper measurements **(e)**. Data from one mouse experiment. *n* = 5 mice per group. *In vivo* data displayed as mean ± SD. *In vitro* data displayed as mean + SD. *P ≤ 0.05 by paired *t*‐tests with Bonferroni correction (*n* = 3 individual donors).

CAR T cells rely on target antigen expression for tumor recognition and clearance. Therefore, any malignant cells without the respective antigen can escape the therapy, culminating in relapse. Unlike CAR T cells, NK cells can recognise malignancies via a range of diverse receptors and are less reliant on singular antigens. To investigate this, NK cells or CD19‐targeting CAR T cells were cultured *in vitro* with parental Raji or CD19^−/−^ Raji cells. Without CD19 expression, Raji cells completely escaped CAR T cells, while NK cells were indifferent to the presence/absence of CD19 and lysed both variants (Figure 8a).

While NK cells displayed clearance of antigen‐negative cells, this assay was limited to a single *in vitro* timepoint and did not accurately reflect the kinetics of relapse during a treatment time course. To model antigen‐negative relapse *in vivo*, NSG mice were engrafted with a mixed culture of 7.5 × 10^5^ CD19^+/+^ and 2.5 × 10^5^ CD19^−/−^ Raji‐Luc cells. The CD19^−/−^ Raji line was engineered to simultaneously express RFP for fluorescent tracking with the IVIS, while the cumulative tumor burden of both lines was quantified through luciferase readouts and physical calliper measurements. For effective imaging of RFP^+^ tumors, Raji cells were subcutaneously injected to avoid the autofluorescence of haemoglobin. Following engraftment, mice were either sequentially treated with 2 × 10^6^ NK cells on Day 2 and 4 × 10^6^ CAR T cells on Day 4, treated with CAR T cells alone or treated with PBS (Figure 8b).

Following engraftment, those mice treated with PBS or a monotherapy of CAR T cells experienced steady growth of tumor burden characterised by increasing luciferase intensity and tumor volumes (Figure 8c–e). At these early stages, the fluorescence of RFP was below the limit of IVIS bioimager detection. When mice were treated with the combination therapy, total tumor burden reduced between Days 20 and 27 (Figure 8c–e) but coincided with the emergence of an RFP^+^ population from Days 27 onwards (Figure 8f and g). Following Day 27, the RFP intensity continued to increase alongside relapsed tumor progression (Figure 8d and e). Flow cytometry on harvested tumors further confirmed that the co‐administered NK cells did not control the outgrowth of antigen‐negative tumors (Supplementary figure 8).

## Discussion

A leukapheresis product is a finite resource. It is therefore imperative to maximise yield from the product within the manufacturing window. This study demonstrates how neglected non‐T‐cell PBMC can be ‘recycled’ into NK cells to improve existing CAR T treatments. By applying feeder cells to a waste product, NK cells are rapidly expanded, with a highly enriched product available in 10 days. In this sense, NK cells can be applied as a pretreatment during CAR T cell manufacture. We showed that pre‐treating with recycled NK cells significantly enhances tumor clearance and survival in breast cancer, acute lymphoblastic leukaemia and non‐Hodgkin's lymphoma xenograft models.

While we improved NK cells through transduction and CAR expression, generating separate CAR NK and CAR T products in parallel would incur additional regulatory measures and increased costs of lentiviral vectors. It is therefore encouraging that recycled NK cells in their native, untransduced form can provide marked improvements to the existing CAR T cell therapies. This pre‐existing cytotoxicity can be linked to the expression of various activation receptors, including NKG2D and NKp30,44 and 46.^20^

Previous use of autologous, untransduced NK cells has yielded disappointing results, with transferred NK cells being largely dysfunctional.^13^ However, the authors noted that these NK cells were likely lacking IL‐2 for full anti‐cancer activity. We highlighted that CAR T cell activity provides NK cells with IL‐2, IFN‐γ and TNF for enhanced anti‐tumor effects above culture in IL‐2 alone. Likewise, T‐cell‐derived factors have been shown to induce aerobic glycolysis for increased NK cell activation and proliferation.^26^, ^27^, ^28^ For *in vivo* experiments, the immunosuppressed NSG mouse model was employed which would limit the availability of gamma‐chain cytokines and contribute to NK cell dysfunction. Indeed, a monotherapy of NK cells displayed no tumor control. Therefore, any improvements conferred from NK to CAR T cells are likely reliant on basal CAR T cell activity. This was confirmed as decreasing the dose of CAR T cells to sub‐effective levels ablated all benefits of the combination therapy (Supplementary figure 9). In contrast, pre‐conditioned CAR T cell patients have elevated gamma‐chain cytokines that might support the activity and persistence of NK cells.^29^

By sampling lymphocytes at the endpoint of a solid‐tumor model, we identified that NK cells promoted the differentiation of T cells into effector memory states, while preventing their terminal differentiation. As the combination therapy was further enhanced through CAR expression on the NK cells, it was considered that the NK cells directly aided in reducing the tumor burden for more durable responses. However, co‐administered NK cells could not suppress antigen‐negative relapse of tumors, indicating that the cytolytic activity of the NK cells may decrease across the time course of treatment. This would be consistent with clinical and experimental observations in which NK cells persist, though are metabolically dysfunctional and do not prevent relapse.^12^, ^30^

To improve this combination therapy, CAR T cells could be modified to ectopically express gamma‐chain cytokines, such as IL‐15 or IL‐21 to benefit both NK and CAR T cells.^31^, ^32^, ^33^ In addition, as the NK cells express high levels of CD16, a tumor‐targeting IgG_1_ could enhance NK‐mediated cytotoxicity.^34^ However, with CD16 rapidly lost following target engagement, even under CAR T‐derived cytokine conditions, the functional relevance of ADCC by expanded NK cells *in vivo* is unclear.

While NK cells are commonly administered as allogeneic cell products, this study involves NK cells expanded in parallel to avoid possible rejection of co‐administered autologous CAR T cells. However, in our cell line‐based experiments, the two cell types were necessarily allogeneic with respect to the orthotopic tumor cells. Autologous NK cells possess pre‐determined KIR alleles that will react variably to cancer ligands depending upon patient HLA allotypes.^35^ Additionally, individuals can possess KIR3DS1^+^ NK cells that react to HLA‐F on activated T cells.^36^ Ideal NK cell products would possess mismatched KIR alleles to enhance graft vs leukaemia effects while lacking detrimental KIRs that could lead to CAR T cell depletion.^37^ ‘Memory’ NK cells (NKG2C/CD57^+^) could also improve the therapy because of their heightened cytotoxicity and longevity.^38^ Biobanks of NK cells expressing libraries of allotypes would be ideal to feasibly and equitably supplement CAR T cells in this setting.

In conclusion, this study has highlighted how autologous NK cells can be expanded alongside CAR T cells for an effective combination therapy of blood and solid cancers. Future studies could determine whether allogeneic NK cells provide similar benefit to co‐administered CAR T cells.

## Methods

### Vector construction

The human PD‐L1 (NCBI 29126) gene with endogenous signal sequence was synthesised by Twist Biosciences and cloned into *SfiI* sites of pSBbiP—a modified pSBbiGP encoding puromycin resistance but with the original GFP gene removed.^39^

The disulphide‐trapped HLA‐E construct (with H1N1 signal sequence) was synthesised as a single‐chain trimer^40^ encoding the cytomegalovirus UL40 peptide (VMAPRTLFL), human β2M (NCBI 567) and human HLA‐E (NCBI 3133) genes, separated with glycine‐serine linkers. The HLA‐E SCT was cloned into *SfiI* sites of pSBbiP.

The B7‐H3‐targeting CAR with modified IL‐2 signal sequence (no.: 104349; Addgene),^41^ was constructed by linking the V_L_ and V_H_ regions of 8H9 IgG^19^ with a (G4S)_3_ linker and cloned into *NcoI* and *NheI* sites of the lentiviral pCCL Sin plasmid.^31^ The CD19 and CD20‐targeting CARs were generated by cloning FMC63 scFv and 1F5 scFv into the CD28‐CD3 based CAR backbone in pCCL‐SIN.PPT together with packaging vectors pMD2.VSVg, pMDLgpRRE and pRSV‐Rev (kind gifted by Luigi Naldini, Fondazione Telethon, Milan, Italy).

### Generation and culture of cell lines

MCF‐7 cells (ATCC HTB‐22; Manassas, VA, USA) were stably transfected with 2 μg of pSBbi plasmid and 0.5 μg of pCMV(CAT)T7‐SB100 transposase (34879; Addgene, Watertown, MA, USA) complexed with lipofectamine 3000 (L3000075; Thermo Fisher) reagents. Media was replaced 16 h after transfection, and transfectants were selected with 1 mg/mL Zeocin (ant‐zn‐5p; InvivoGen, San Diego, CA, USA) or 2 μg/mL Puromycin (ant‐pr‐5; InvivoGen) for PD‐L1 and HLA‐E SCT transfectants, respectively.

MCF‐7 cells transfected with PD‐L1, full‐length Her2 (183246; Addgene) and luciferase^31^ were cultured in high‐glucose Dulbecco's modified Eagle Medium (12100‐046; Gibco/Thermo Scientific, Waltham, MA, USA), supplemented with 10% inactivated foetal calf serum (FCS; C8056; Sigma‐Aldrich, Burlington, MA, USA) at 37°C with 5% CO_2_.

Artificial antigen presenting cells (aAPC) expressing 4‐1BBL, CD86 and membrane‐bound (mb)‐IL‐15 and mb‐IL‐21 were generated by transfecting K562 (ATCC CCL‐243) with Sleeping Beauty vectors.^31^

Likewise, NALM6‐luc and Raji‐luc cells were generated through Neon electroporation and transfection with luciferase‐encoding Sleeping Beauty vectors and selected through puromycin resistance.

aAPC, NALM6‐luc and Raji‐luc were maintained in Roswell Park Memorial Institute‐1640 (31800‐022; Gibco/Thermo Scientific) supplemented with 10% FCS (R10). aAPC were irradiated at 100Gy prior to cryopreservation in 10% dimethyl sulfoxide (DMSO; D2650‐100ML; Sigma‐Aldrich), 90% FCS.

### Parallel expansion of NK cells and CAR T cells

Peripheral blood mononuclear cells (PBMC) were isolated from the blood of consenting, healthy donors (ethics approval number: H18/089). Primary cells were cultured in R10 supplemented with 50 IU/mL interleukin‐2 (IL‐2; 200‐02; PeproTech, Rocky Hill, CT, USA) for all steps and incubated at 37°C with 5% CO_2_.

To generate CAR T cells, T cells were isolated using a negative‐selection kit (17951; STEMCELL Technologies, Vancouver, Canada) and activated with CD3/CD28 Dynabeads™ (11132D; Gibco/Thermo Scientific) for 48 h. T cells were spinoculated onto LV‐retronectin coated 24‐well plates using LV MOI 20 then incubated for 16 h prior to Dynabead removal and replacement of culture medium. T cells were further subcultured at 1 × 10^6^ cells/mL for 10 days in 24‐well plates.^31^

During T cell isolation, residual PBMC (non‐T PBMC) were immobilised on the sides of the isolation tube. Following removal of the magnetic field, the non‐T PBMC were aspirated with a gentle media wash and seeded at 1:1 with 100 Gray‐irradiated aAPC at a total cell concentration of 1.25 × 10^5^ cells/mL in a vertically standing T75 flask to a final volume of 40 mL of R10 with 50 U/mL IL‐2. Half of the media was replaced with fresh R10 on Days 3 and 5, with IL‐2 concentration replenished for the entire volume. NK cell cultures were subsequently split 1:1 on Day 7 and then split 1:3 every other day to allow optimal expansion.

### Cytotoxicity assays

To assess cytotoxicity, MCF‐7 cell lines were seeded at 5 × 10^5^ cells/mL in 96‐well plates (100 μL/well). After 16 h of incubation, 50 μL of supernatant was removed and replaced with 50 μL of effector cells to achieve respective effector‐to‐target ratios. Co‐cultures of effector and target cells were incubated for a further 24 h. Following incubation, 50 μL of cell medium was removed and replaced with 50 μL of Firefly Luc One‐Step Substrate (1862810; Thermo Fisher) diluted 1/100 in Firefly Luc One‐Step Assay Buffer (1862812; Thermo Fisher). The plate was then incubated at room temperature, in the dark, for 40 min before reading bioluminescence with a Varioskan LUX multimode microplate reader (Thermo Fisher). Cytotoxicity was calculated by comparing luciferase emission signals of effector:target co‐cultures to that of tumor cells only.

During ADCC assays, tumor cells were coated with 10 μg/mL avelumab for 1 h, prior to gentle washing of supernatant and subsequent addition of effector cells. Avelumab was produced in ExpiCHO‐S cells as previously described.^42^

For Raji cytotoxicity assays, target cells were incubated with NK cells at a 1:1 ratio for 24 h prior to flow cytometric analysis. Co‐cultures were stained for CD3, CD56 and CD20. A set volume of 75 μL was acquired and CD20^+^ events were enumerated and compared to a target‐cell‐only control.

### Activated CAR T and NK cell supernatant analysis

#### Preparation of activated CAR T cell supernatant

To analyse the constituents of activated CAR T and NK cell supernatant, CAR T cells (2.5 × 10^5^) were seeded at a 1:1 ratio with MCF‐7 cells overexpressing PD‐L1, HER2 and luciferase and incubated in RPMI supplemented with 10% FCS at 37°C and 5% CO_2_ for 24 h. IL‐2 was excluded from the culture medium.

Following incubation, the supernatant was isolated through differential centrifugation at 450 RCF for 5 min. The resulting supernatant was further centrifuged at 2000 RCF for 20 min before being passed through a 0.2‐μm filter to ensure minimal vesicle and cellular contamination.

#### IL‐2 ELISA

Isolated CAR T and NK cell supernatants were subjected to sandwich ELISA for IL‐2 analysis. In brief, wells of a 96‐well maxisorb plate were coated with 2 μg/mL purified mouse anti‐human IL‐2 (BD555051; Becton Dickson) diluted in phosphate‐buffered saline (PBS) and incubated at 4°C overnight. Following incubation, the wells were washed three times with 0.05% Tween 20 diluted in PBS before the wells were blocked with 200 μL of 1% BSA/PBS for 10 min at room temperature. After 10 min of blocking, the BSA/PBS was removed and replaced with either 100 μL of supernatant or samples for standard curve generation. To generate a standard curve, a serial dilution of recombinant huIL‐2 (Peprotech) diluted in RPMI was included. As a negative background control, wells including RPMI only were included. Supernatants and samples were incubated at 37°C for 1 h, before removal and washing of plates. After washing, 100 μL of 1 μg/mL biotinylated anti‐human IL‐2 (BD555040) was added to the samples, and the plate was incubated at 37°C for 1 h before washing and the addition of streptavidin horse radish peroxidase complex, diluted 1/5000 in 1% BSA/PBS and a further 1 h incubation at 37°C. After a final wash step, 100 μL of TMB + 1/2000 H_2_O_2_ was added to the wells and the plate was allowed to develop at room temperature in the dark. To disrupt development, wells were added with 50 μL of 1 N H_2_SO_4_ and the plate was read at 450 nm in a Varioskan LUX multimode microplate reader.

#### Mass spectrometric analysis

Proteins were enriched from cell culture supernatant and digested with trypsin using the filter‐aided sample preparation FASP method.^43^ Digested samples were then analysed by liquid chromatography coupled tandem mass spectrometry (LC–MS/MS) using a Neo Vanquish nanoflow LC system coupled to an Orbitrap Exploris 480 mass spectrometer (Thermo Scientific) operated in data‐dependent acquisition mode (DDA). Data analysis was performed with the Proteome Discoverer software (Version 3.2; Thermo Scientific) for protein identification and quantification.

### Xenograft models

For engraftment of human lymphocytes and tumors, NOD‐*scid* IL2Rgamma^null^ (NSG) mice (005557; Jackson Laboratory, Bay Harbor, ME, USA) were bred within the University of Otago Eccles Building under specific pathogen‐free conditions. The study was approved by the University of Otago Animal Ethics Committee under a respective animal use protocol. For solid‐tumor experiments, mice were subcutaneously injected in the ventral lateral flank with 2 × 10^6^ MCF‐7 cells (overexpressing PD‐L1, HER2 and luciferase). Mice were then intravenously injected with 5 × 10^6^ NK cells and 2.5 × 10^6^ CAR T cells on Days 10 and 12 post tumor engraftment, respectively. One tumor group was allocated PBS injections as controls. For blood‐borne malignancies, mice were intravenously engrafted with either 1 × 10^6^ NALM6‐luc or Raji‐luc on Day 0, prior to 4 × 10^6^ NK cells on Day 2 and 2.5 × 10^6^ CAR T cells on Day 4.

To minimise confounding factors, all mice were housed in the same room and treated simultaneously. Following tumor engraftment, mice were randomly assorted into treatment/control groups. Investigators were aware of group allocation at all stages of the experiment. A sample size of four to five mice per group ensured sufficient statistical power while streamlining workflow for bioimaging. In the solid‐tumor experiment, one mouse in the PBS group was removed from the study due to insufficient tumor engraftment (< 1 × 10^8^ photons/s on Day 7 post‐engraftment). In the NALM‐6 experiment, one mouse in the NK + CAR T group was removed from the experiment on Day 42 because of a non‐tumor‐related event and was censored from future statistics.

Tumor burden was monitored through weekly live imaging with the PerkinElmer IVIS X5 system. Mice were intraperitoneally injected with 150 mg/kg D‐luciferin potassium salt (LUCK‐1G; GoldBio, Saint Louis, MO, USA) and anaesthetised with isoflurane. Mice were imaged either 6 or 12 min post‐injection on auto‐exposure settings for haematological and solid malignancies, respectively. Five images were taken with 1‐min intervals. Images were analysed with the Living Image software (PerkinElmer) and the bioluminescent signal flux was expressed as photons/second.

Solid tumors were also monitored every second day with digital callipers. Volume was calculated as 0.5 × length × width^2^. Mice were euthanised when solid‐tumor volumes reached 1000 mm^3^. Mice in all experiments were monitored daily for behavioural changes and were euthanised upon hind‐limb paralysis and/or weight loss of 20%.

### Flow cytometry

To label for surface proteins, cells were incubated in FACS wash buffer (0.5% bovine serum albumin/2 mM ethylenediaminetetraacetic acid/PBS) with antibodies for 15 min on ice. PD‐L1 expression on MCF‐7 cells was confirmed by staining cells with avelumab and subsequently staining with anti‐human IgG.

At the end of select *in vivo* studies, spleens and tumors were removed from euthanised mice for flow cytometric analysis. Tumors were mechanically dissociated and enzymatically digested through a 60‐min incubation in 1 mg/mL collagenase type I and 200 U/mL DNAse at 37°C on a rotator to generate a single cell suspension. Spleens were mechanically dissociated through 70‐μm cell strainers to generate single cell suspensions. Single cell suspensions were washed and incubated in 100 μL red blood cell lysis (ACK) buffer for 1 min prior to the addition of 1 mL FACS wash buffer and centrifugation. Resuspended cells were then subjected to antibody staining for flow cytometry.

Data were acquired with a Cytek Aurora with the SpectroFlo 3.3.0 software (Cytek Biosciences, Fremont, CA, USA). Data were analysed with the FlowJO version 10.10.0 software. Events were subjected to forward‐ and side‐scatter doublet discrimination with dead cells excluded with Zombie NIR viability dye. Donor and variable‐specific unstained controls were employed for accurate autofluorescence removal during unmixing. High‐dimensional analysis was carried out with OMIQ (Dotmatics; Boston, MA, USA), including Flow Self Organising Map (SOM) meta clustering and Uniform Manifold Approximation and Projection (UMAP) analysis and figure generation.

The monoclonal antibodies used in this study are as follows: anti‐human CD3 Alexa Fluor (AF) 700 (1 μg/mL; 300424; BioLegend, Sand Diego, CA, USA), anti‐human CD56 allophycocyanin (0.25 μg/mL; 318310; BioLegend), anti‐human CD56 Brilliant Violet (BV) 421 (0.12 μg/mL; 318327; BioLegend), anti‐human CD16 BV711 (0.50 μg/mL; 302043; BioLegend), anti‐human TIM‐3 BV650 (1 μg/mL; 565564; BD Biosciences, Franklin Lakes, NJ, USA), anti‐human TIGIT BV605 (1 μg/mL; 747841; BD Biosciences), anti‐human NKG2A AF647 (0.25 μg/mL; 375106, BioLegend), anti‐G4S phycoerythrin (1 μg/mL; 38907S; Cell Signalling Technology, Danvers, MA, USA), anti‐human HLA‐E allophycocyanin (1 μg/mL; 342606; BioLegend), anti‐human IgG AF647 (0.5 μg/mL; 109‐605‐003; Jackson Immuno Research, West Grove, PA, USA), anti‐human NKG2D PE‐Cy7 (0.5 μg/mL; BIO320811; BioLegend), anti‐human NKG2C PE (1 μg/mL; 375003; BioLegend), anti‐human NKp30 BUV395 (4 μg/mL; 743173; BD Biosciences), anti‐human NKp44 BUV615 (8 μg/mL; 752353; BD Biosciences), anti‐human NKp46 APC/Fire 750 (1 μg/mL; BIO331933; BioLegend), anti‐human CD16 FITC (0.4 μg/mL; BIO302005; BioLegend), anti‐human CD57 BV785 (0.0625 μg/mL; 393329; BioLegend), anti‐human TIGIT PerCP/Cy5.5 (0.25 μg/mL; BIO372717; BioLegend), anti‐human CD19 BV510 (0.25 μg/mL; 302242; BioLegend) and anti‐human CD20 APC‐Cy7 (1 μg/mL; 302313; BioLegend).

### Data analysis and statistics

Schematic illustrations were made using BioRender and Powerpoint. All *in vitro* experiments were repeated with at least three unique donors. Data are presented as mean ± standard deviation or median ± interquartile range. Representative flow cytometry plots are from one donor. Paired *t*‐tests, two‐way analysis of variance (ANOVA) and log‐rank (mantel‐cox) tests were used to determine statistical significance. Statistical analysis and graphs were generated and validated within GraphPad Prism 10 software. *P*‐values < 0.05 were determined as statistically significant. * = *P* ≤ 0.05, ** = *P* ≤ 0.005, *** = *P* ≤ 0.0005, **** = *P* < 0.0001.

## Author contributions

**Thiloma D Liyanage:** Investigation; data curation. **Patrick W Marron:** Investigation; data curation. **Clare Y Slaney:** Resources; methodology. **Alexander D McLellan:** Conceptualization; investigation; funding acquisition; writing – review and editing; writing – original draft; methodology; supervision; resources; project administration. **Ariana HT Drabble:** Investigation; data curation. **Kristine C Pe:** Investigation; data curation. **Barry D Hock:** Conceptualization; methodology; supervision. **Lachlan J Dobson:** Conceptualization; investigation; writing – original draft; methodology; writing – review and editing; formal analysis; data curation.

## Conflict of interest

The authors declare no conflicts of interest.

## Ethical approval

This study was approved by the University of Otago Human Ethics Committee (H18/089). All participants provided informed, written consent.

## Consent for publication

Not applicable.

## Supporting information


Supplementary figure 1
Supplementary figure 2
Supplementary figure 3
Supplementary figure 4
Supplementary figure 5
Supplementary figure 6
Supplementary figure 7
Supplementary figure 8
Supplementary figure 9

## Data Availability

All data relevant to the study are included in the article and accompanying Supplementary material.
