# Supplementary material for: Parallel processing of T cells and natural killer cells to enhance in vivo CAR T cell activity against blood and solid cancers
Source: Clin Transl Immunology. 2026 Sep 1;15(9):e70120. doi: 10.1002/cti2.70120 (PMC13534565; doi:10.1002/cti2.70120)
Supplement: Supplementary file 1 [file CTI2-15-e70120-s001.docx]

**Supplementary Figures**

**Supplementary Figure 1.** **CD56 and CD16 expression following aAPC exposure.** Following T cell isolation, the remaining non-T PBMC were exposed to aAPC for simultaneous NK cell expansion. Non-T PBMC were stained for CD3, CD56 and CD16 before (day 0) and following aAPC exposure. Above flow plots were pre-gated on CD3-live cells. Data displays three individual donors on days 0, 2 and 6.

**Supplementary Figure 2. NKG2A and NKG2C expression pre- and post-aAPC exposure.** NK cells were expanded for 14 days and subjected to flow cytometry on days 0, 7 and 14. NK cells were initially gates as CD3^-^, CD56^+^ lymphocytes and subsequently assessed for NKG2A and NKG2C expression (a-b). The frequency of NKG2A^+^ and NKG2C^+^ NK cells pre- and post-expansion were compared, revealing no significant changes in either population (c). Flow cytometry plots display the same representative donor tracked across the expansion period. Data is displayed as individual donors (b) and as mean ± SD (c). n = 4 individual donors. Means were compared through multiple paired T-tests, followed by Bonferroni corrections. (ns = p > 0.05).

**Supplementary Figure 3. Identification of cytokines and chemokines in the activated CAR T cell supernatant.**
CAR T cells were activated through overnight co-culture with PD-L1^hi^, HER2^hi^ MCF-7 cells at a 1:1 effector-to-target ratio. Supernatant was collected following incubation and subjected to mass spectrometric analysis. Proteins in the supernatant were identified and quantified with Proteome Discoverer software and ranked based on the cumulative ion counts from four individual CAR T cell donors. Relevant cytokines and chemokines have been highlighted.

**Supplementary Figure 4. Identifying a non-curative dose of CAR T cells.** NSG mice were engrafted with Her2^hi^ **/** PDL1^hi^ MCF-7 cells on day 0 prior to CAR T cell administration on day 12. Mice were treated with a titration of CAR T cell doses and imaged weekly to monitor tumour burden. (n = 6 mice per group. Tumour radiance displayed as mean) . Data from one mouse experiment.

**Supplementary Figure 5. Impact of the timing of NK cell administration**. NSG mice were engrafted with Her2^hi^ / PD-L1^hi^ MCF-7 cells on day 0 prior to NK cell infusion on either days 10 or 12. CAR T cells were infused on day 12 in both conditions. (a) Displays the tumour burdens over time while (b) displays the burden at day 35. Data is representative of two separate NSG experiments. n = 4-6 mice per group. Time course data displayed as median. Day 35 data displayed as mean + SD and analysed by two-way ANOVA with Bonferroni corrections where * = P < 0.05 and ** = P < 0.01.

**Supplementary Figure 6. Combination therapy against MCF-7 cells with parental PD-L1 expression levels.**
NSG mice were engrafted with MCF-7 cells (WT PD-L1 expression) on day 0 prior to administration of NK cells on day 20 and CAR T cells on day 22 (a). Tumours were monitored weekly through bioluminescent imaging (b, c). Data from one mouse experiment; n = 4 mice per group. Data displayed as mean ± SEM.

**Supplementary Figure 7. Expressing CAR in NK cells.** The EF1a and MSCV promoters were compared for transduction frequency and CAR expression in NK cells. NK cells were transduced on day 7 of expansion at an MOI of 5. Summary data is shown in (A) with a representative flow plot from the MSCV promoter in (B). (Data displayed as mean + SD. n=3 individual donors, ** = p ≤ 0.01 by Two-way ANOVA and Bonferroni correction).

**Supplementary Figure 8. Tumour cell phenotypes at ethical endpoint.**
Mice were euthanised when subcutaneous Raji tumours reached 1000mm^3^_._ Following euthanasia, tumours were excised, processed, and analysed with flow cytometry. The tumours treated with a monotherapy of CAR T cells resembled wildtype (CD19^+^, RFP^-^), while the tumours treated with the combination therapy were dominated by CD19^-^, RFP positive cells. Flow plots resemble one representative tumour from each respective condition.

**Supplementary Figure 9. A non-tumour-clearing dose of CAR T cells ablates benefits of combination therapy.** NSG mice were engrafted with PDL1^hi^ MCF-7 cells on day 0 prior to NK and CAR T cell administration on days 10 and 12, respectively. Mice were treated with 2×10^6^ NK cells and 1×10^6^ CAR T cells and imaged weekly to monitor tumour burden. (n = 5 mice per group. Tumour radiance displayed as mean ± SEM). Data from one mouse experiment.
